# Dioxins and the One Health Paradigm: An Interdisciplinary Challenge in Environmental Toxicology

**DOI:** 10.3390/toxics13110964

**Published:** 2025-11-07

**Authors:** Marília Cristina Oliveira Souza, Jose L. Domingo

**Affiliations:** 1 Department of Biomolecular Sciences, School of Pharmaceutical Sciences of Ribeirao Preto, University of Sao Paulo, Av. do Café s/n, Ribeirao Preto 14040-903, Sao Paulo, Brazil; mcosouza@usp.br; 2 Laboratory of Toxicology and Environmental Health, School of Medicine, Universitat Rovira i Virgili, Sant Llorenç 21, 43201 Reus, Catalonia, Spain

**Keywords:** dioxins, one health, environmental toxicology, biomagnification, climate-dioxin interactions, planetary health

## Abstract

Dioxins are legacy and persistent environmental pollutants that pose complex and far-reaching risks to human, animal, and ecosystem health. As unintentional byproducts of industrial and combustion processes, dioxins accumulate in the environment, biomagnify through food webs, and exert toxic effects even at low concentrations. This review applies a One Health lens to synthesize current knowledge on dioxin sources, environmental fate, exposure pathways, and toxicological impacts across species. We have critically examined existing surveillance systems, regulatory frameworks, and policy responses, highlighting both achievements and persistent gaps. A fully integrated One Health approach, linking environmental, animal, and human health domains, is essential for effective monitoring, risk assessment, and mitigation. It includes cross-sectoral collaboration, harmonized biomonitoring, evidence-based policy interventions, and transparent risk communication. Emerging evidence on climate-driven dioxin remobilization and microplastic interactions further underscores the urgency of adaptive, system-based strategies. Strengthening global capacity through such integrative approaches is vital to safeguard planetary health from these enduring contaminants. Quantitative insights and illustrative examples support these conclusions.

## 1. Introduction

Dioxins are a group of highly persistent environmental pollutants, which include polychlorinated dibenzo-*p*-dioxins (PCDDs), polychlorinated dibenzofurans (PCDFs), and dioxin-like polychlorinated biphenyls (dl-PCBs). Dioxins represent a significant subgroup of persistent organic pollutants (POPs), distinguished by their remarkable environmental persistence, tendency to bioaccumulate, and considerable toxic potential [1,2,3]. These pollutants are well-known for their ability to accumulate in fatty tissues, undergo biomagnification throughout food chains, and exert significant toxic effects even at very low concentrations [4,5]. This combination of traits makes them a critical concern for both environmental and human health [6,7,8,9]. Their ubiquitous presence stems largely from anthropogenic activities, including waste incineration, chemical manufacturing (particularly involving chlorine), metal smelting, and historical pesticide production [10]. Once released, dioxins enter ecosystems, bind strongly to soil and sediments, and cycle through environmental compartments, leading to widespread, low-level background exposure for most organisms, including humans, primarily via the food chain [10,11,12,13].

The Stockholm Convention identifies dioxins as priority pollutants requiring urgent action, with recent amendments targeting dl-PCBs from informal e-waste recycling [14]. Moreover, recent studies have highlighted the role of climate change in altering dioxin dynamics, with projections indicating an increase in secondary emissions from thawing permafrost by 2050 [15,16,17,18].

Addressing the complex challenges posed by dioxins necessitates an integrated perspective, such as that offered by the One Health concept [19]. Recognizing that the health of humans, domestic and wild animals, and the wider environment are inextricably linked, One Health promotes collaborative, multisectoral, and transdisciplinary approaches [20,21,22,23]. In this sense, the One Health framework, endorsed by the Quadripartite Alliance (FAO, UNEP, WHO, WOAH), is critical for effectively addressing dioxin contamination. Its effectiveness has been demonstrated in managing cross-sectoral crises such as antimicrobial resistance and zoonotic pandemics [24,25]. This framework is particularly apt for contaminants like dioxins, whose sources, transport, exposure pathways, and effects inherently cross environmental, animal, and human domains [20,26]. This approach enables a comprehensive understanding of multi-pathway exposure dynamics (e.g., soil–plant–livestock–human transmission) and interspecies health impacts (e.g., parallel endocrine disruption patterns in wildlife and human populations), fostering integrated risk analysis and cross-sectoral policy frameworks that bridge disciplinary divides [27,28]. Recent outbreaks of dioxin contamination in subsistence fishing communities [29] exemplify the intersection of cultural, ecological, and health vulnerabilities central to a One Health framework.

This review aims to synthesize current knowledge of dioxin contamination through an explicit One Health perspective. By integrating evidence across disciplines, we highlight pathways of exposure, ecological and human health effects, and policy innovations, culminating in recommendations for future integrative surveillance and risk management strategies.

## 2. Methods

### 2.1. Search Strategy

This review has employed a methodology designed to synthesize current knowledge on dioxin contamination through an explicit One Health lens. The approach combined literature search strategies with integrative analysis to examine the interconnected dimensions of environmental, animal, and human health impacts of dioxin exposure. The literature search was conducted across Web of Science (https://www.webofscience.com), PubMed (https://pubmed.ncbi.nlm.nih.gov), and Scopus (https://www.scopus.com), covering publications from database inception through 31 August 2025. The search strategy employed both individual and combined keyword searches using Boolean operators (AND, OR) to capture relevant literature.

Search terms included “dioxins”, “polychlorinated dibenzo-p-dioxins (PCDDs)”, “polychlorinated dibenzofurans (PCDFs)”, “One Health”, “environmental toxicology”, “bioaccumulation”, “biomagnification”, “human health effects”, “animal health”, “wildlife toxicology”, “ecosystem health”, “aryl hydrocarbon receptor (AhR)”, “Toxic Equivalency Factor (TEF)”, “dioxin sources”, “dioxin regulation”, “climate change and dioxins”, and “microplastics and dioxins”. Search combining “One Health” with dioxin-related terms yielded limited results, highlighting a significant research gap in interdisciplinary approaches to this environmental health challenge. It guided our synthesis approach toward identifying opportunities for enhanced integration across health domains.

Inclusion criteria encompassed peer-reviewed research articles, systematic reviews, meta-analyses, government and international agency reports (WHO, UNEP, EFSA, US EPA), regulatory guidelines, and technical documents published in English. No temporal restrictions were applied. Data extraction focused on: (1) environmental sources and fate of dioxins, (2) toxicological mechanisms across species, (3) human health impacts, (4) wildlife and ecosystem effects, (5) surveillance and monitoring approaches, (6) regulatory responses, and (7) interdisciplinary management strategies. Information was synthesized thematically using the One Health framework as an analytical lens to identify cross-domain connections, knowledge gaps, and opportunities for integrated approaches.

Critical analysis involved examining consistencies and discrepancies across studies, identifying methodological limitations, and highlighting emerging research directions. Special attention was given to examples demonstrating interconnections between environmental contamination, wildlife health impacts, and human exposure pathways, consistent with One Health principles.

### 2.2. One Health Framework Application to Dioxin Contamination

The One Health paradigm is particularly relevant to dioxin contamination because exposure pathways are fundamentally interconnected across domains. For example, dioxin contamination in agricultural soils directly affects crop uptake, which then impacts livestock through feed consumption, reaching humans through consumption of animal-derived products (meat, dairy products, eggs). Similarly, aquatic contamination affects fish populations, which serve as both wildlife food sources and human dietary components, creating shared exposure pathways between wildlife and human populations, especially in subsistence communities.

This interconnectedness means that addressing dioxin contamination requires coordinated surveillance across environmental media, wildlife sentinel species, agricultural systems, and human populations. While traditional approaches miss critical exposure pathways and fail to recognize early warning signals that could prevent widespread contamination events, the One Health framework provides the conceptual foundation for such integrated approaches.

**Figure 1:**
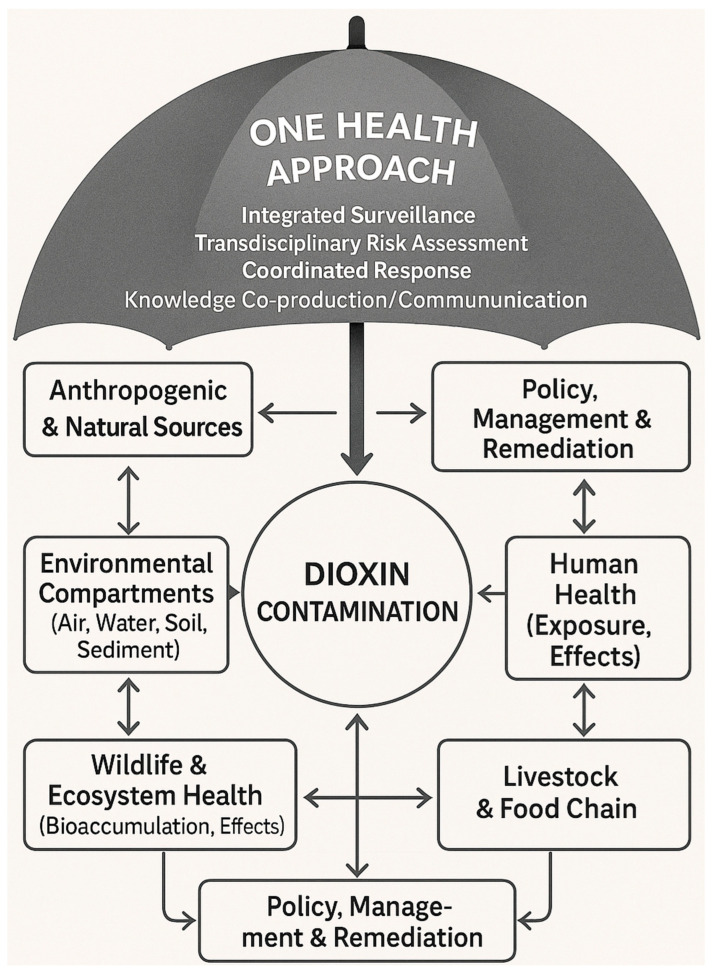
Systems-based diagram illustrating the interconnected pathways of dioxin contamination. Primary sources (e.g., incineration, e-waste recycling), environmental transport through air, water, and soil, impacts on wildlife (e.g., reproductive failure in seabirds), and human health outcomes (e.g., carcinogenicity) are depicted. Arrows highlight interdependencies between these components, indicating the need for integrated surveillance and coordinated policy responses within a One Health framework.

Figure 1 illustrates the interconnected pathways through which dioxins impact environmental, animal, and human health systems. It is shown how anthropogenic sources (industrial processes, waste incineration, e-waste recycling) release dioxins into environmental matrices (air, water, soil), where they undergo long-range transport and bioaccumulation. These environmental reservoirs then serve as exposure sources for both wildlife populations and human communities through multiple pathways.

## 3. Environmental Sources, Fate, and Distribution of Dioxins

Dioxins are not intentionally produced but are formed as unwanted byproducts. Major anthropogenic sources include:

**Waste Incineration**: Combustion of municipal, medical, and hazardous waste, particularly under suboptimal conditions [30,31,32].

**Industrial Processes:** Metallurgical processes (e.g., smelting, sintering), cement kilns, chemical manufacturing (e.g., chlorinated phenols, herbicides like 2,4,5-T, PVC production) [33,34,35].

**Combustion**: Burning of fossil fuels (coal, oil), wood (especially treated wood), and biomass, including uncontrolled landfill fires [36,37].

**Pulp and Paper Industry**: Chlorine bleaching processes (though largely phased out in many regions in favor of elemental chlorine-free (ECF) or totally chlorine-free (TCF) methods) [38,39].

**Natural Sources**: Forest fires and volcanic eruptions contribute but are generally considered minor compared to anthropogenic inputs globally, though potentially significant locally or regionally [40,41].

**Reservoir Sources**: Contaminated soils and sediments act as long-term reservoirs, with potential for remobilization [42,43].

**E-Waste Recycling**: In recent years, the recycling of electrical and electronic waste (e-waste) has emerged as a significant source of dioxins and dioxin-like compounds, particularly in Asian countries [44,45].

**Biomass Combustion**: Residential wood burning in developing nations contributes to dioxin emissions, which are exacerbated by the use of inefficient stoves and contaminated fuel sources [46,47].

**Open Burning**: Uncontrolled open burning of municipal solid waste and agricultural residues is a significant and growing source of dioxin emissions, particularly in Asia and Africa where waste management infrastructure is limited. This practice releases dioxins directly into the local environment, posing immediate health risks to nearby populations and contributing to regional background levels [48].

From a One Health perspective, these diverse sources demonstrate how industrial activities, waste management practices, and energy production decisions create shared environmental exposures that simultaneously affect human populations, domestic animals, and wildlife. For instance, municipal waste incineration near agricultural areas can contaminate both human food supplies and wildlife habitats through the same atmospheric deposition processes, creating exposure pathways that require coordinated monitoring and management approaches.

Since the 1980s and 1990s, regulatory initiatives, particularly the implementation of Best Available Techniques (BAT) and Best Environmental Practices (BEP) under the Stockholm Convention, have significantly curtailed air emissions of POPs in developed nations, achieving very notable reductions in many cases [48]. However, emerging economies undergoing rapid industrialization may still experience increasing emissions if stringent controls lag behind growth [45].

### 3.1. Environmental Fate and Transport

Released primarily into the air, dioxins undergo atmospheric transport and deposition onto soil and water [41,49]. Their hydrophobicity leads to strong partitioning onto organic matter in soils and sediments, resulting in long environmental half-lives (ranging from decades to centuries in some soil types) [42,50]. Sediments act as major sinks but also as potential secondary sources via resuspension [51,52]. Global distillation processes facilitate long-range atmospheric transport to remote regions, such as the Arctic, as recently reviewed by Borgå et al. [53], de Wit et al. [54], and Xie et al. [55].

This environmental transport creates a unified exposure landscape, where contamination from industrial sources can affect the environment and the wildlife and human populations, which depend on them. Arctic communities, for example, experience elevated dioxin exposure despite being far from industrial sources. It indicates how environmental transport mechanisms create shared vulnerabilities across geographic and species boundaries, a key insight that supports the necessity of global, coordinated One Health approaches.

### 3.2. Climate Change and Microplastics as a Vector

Beyond their primary sources, dioxins are increasingly affected by secondary anthropogenic stressors that alter their environmental fate, transport, and bioavailability, impacting ecosystems and human health. Climate change and plastic pollution (microplastics issue) can reshape how legacy dioxins are mobilized, distributed, and transferred through ecosystems. These emerging factors introduce new exposure pathways and complicate risk assessments by accelerating environmental cycling and trophic transfer. Two critical examples of these interactions can be highlighted: the release of dioxins from thawing permafrost and the role of microplastics as vectors of bioavailable dioxins in aquatic systems [56,57,58,59].

- **Permafrost Thaw**: Arctic studies reveal that thawing permafrost releases legacy dioxins stored since the 1970s, increasing concentrations in freshwater [57,58].
- **Microplastic Carriers**: Dioxins adsorbed onto microplastics in marine environments exhibit 50% higher bioavailability to filter feeders, such as mussels, thereby accelerating trophic transfer [56,59].

These climate-driven changes exemplify the dynamic nature of dioxin exposure risks, highlighting how environmental changes can simultaneously impact multiple species and ecosystems. Thawing permafrost releases contaminants into freshwater systems used by both wildlife and human communities, while microplastic-mediated transport affects marine food webs that provide sustenance for both seabirds and coastal human populations.

On the other hand, climate change is an emerging concern that may alter the environmental fate of dioxins. This includes increased extreme weather events (causing erosion and resuspension), melting glaciers releasing legacy contamination, and shifts in soil organic matter dynamics [60,61,62]. Interactions with microplastics in aquatic environments may also influence their transport and bioavailability to organisms.

### 3.3. Soil and Sediment Reservoirs of Dioxins

Dioxins exhibit a strong affinity for organic soil matter, resulting in long-term accumulation in terrestrial environments. Sediments also serve as major sinks for dioxins, but under certain conditions, such as erosion or resuspension, these contaminants can be remobilized into aquatic systems [50,52].

- **Biochar Remediation**: Among remediation strategies, biochar amendment has shown potential to reduce dioxin bioavailability in soils through enhanced sorption. However, the long-term stability of this approach remains an important consideration for environmental management [63,64].

Soil and sediment contamination create long-term exposure reservoirs that affect agricultural productivity, wildlife quality, and human health through multiple pathways. Contaminated agricultural soils affect crop uptake, which impacts both livestock and human food supplies. In turn, polluted sediments affect aquatic ecosystems that support both fisheries and wildlife populations. This persistence presents ongoing One Health challenges that demand sustained and coordinated management strategies.

## 4. Mechanisms of Toxicity

The primary mechanism of toxicity for dioxins and related dl-compounds involves binding to the aryl hydrocarbon receptor (AhR), a ligand-activated transcription factor present in virtually all vertebrate cells [65,66]. The prototypical and most toxic dioxin congener, 2,3,7,8-tetrachlorodibenzo-*p*-dioxin (TCDD), serves as the reference compound for understanding the mechanisms and health impacts of these pollutants [67,68]. Its interaction with the AhR is central to its toxicity, initiating downstream events affecting gene expression and cellular function [69,70]. Central to assessing the risk of complex dioxin mixtures found in the environment is the Toxic Equivalency Factor (TEF) concept, which relates the toxicity of individual congeners to that of TCDD, allowing for the calculation of a total Toxic Equivalence (TEQ) for a mixture [71,72].

The universality of the AhR pathway across vertebrate species provides a mechanistic foundation for the One Health approach to dioxin toxicity. While the basic receptor mechanism is conserved, species-specific differences in receptor sensitivity, metabolic capacity, and downstream responses create varying vulnerability patterns across wildlife and human populations.

**AhR Pathway**: Ligand (dioxin) binding causes the AhR to translocate to the nucleus, dimerize with the ARNT protein (AhR nuclear translocator), and bind to specific DNA sequences called Dioxin Response Elements (DREs) [73,74].

- **Gene Expression Changes**: This binding alters the transcription of a wide array of genes, including those involved in xenobiotic metabolism (e.g., CYP1A1), cell cycle control, development, immune function, and endocrine signaling [73,75].
- **Toxicity**: While induction of metabolic enzymes is an adaptive response, the sustained activation and broad downstream effects triggered by persistent ligands like TCDD lead to cellular dysfunction and toxicity [70,76].
- **TEF Concept**: The TEF system assigns factors to individual dioxins and dl-PCB congeners based on their AhR binding affinity and ability to elicit AhR-mediated responses relative to TCDD (TEF = 1). This allows calculation of a total TEQ for complex mixtures, facilitating risk assessment [72,77].

### 4.1. AhR Signaling Pathways

Activation of the aryl hydrocarbon receptor (AhR) by dioxins triggers a cascade of biological responses with long-term and transgenerational consequences. One critical mechanism involves epigenetic modifications that persist across generations, affecting both detoxification pathways and disease susceptibility.

- **Transgenerational Epigenetics**: TCDD exposure in zebrafish induces DNA methylation changes in CYP1A promoters, which persist for three generations and impair the detoxification capacity [78,79,80]. Additionally, recent studies have demonstrated transgenerational epigenetic effects in mammals, with paternal TCDD exposure altering sperm DNA methylation patterns in mice, linked to metabolic disorders in offspring [81,82,83]. These findings highlight the potential of dioxins to induce transgenerational health effects in both wildlife and humans, remarking the need for a One Health approach to long-term chemical exposure.
- **Non-Canonical Immune Effects**: AhR signaling can modulate immune responses. AhR activation in dendritic cells suppresses IL-12 production, increasing susceptibility to viral infections in mice [84,85]. Such effects show another layer of vulnerability in exposed populations, with possible implications for disease resilience across species. Immune suppression in wildlife populations could increase their susceptibility to emerging infectious diseases, while parallel immune effects in human populations could compromise responses to vaccination programs or seasonal infectious diseases, demonstrating how dioxin exposure creates shared vulnerabilities across the human–animal interface.

### 4.2. Cross-Species Vulnerabilities and One Health Implications

Sensitivity to dioxin exposure varies markedly across species due to differences in AhR structure, ligand-binding affinity, metabolic capacity, and life history traits [69]. For example, guinea pigs are highly sensitive to TCDD, while hamsters are relatively resistant, reflecting variations in AhR-mediated transcriptional responses. Marine mammals, such as cetaceans, present an additional vulnerability due to their lack of functional CYP1A2 enzymes, resulting in prolonged dioxin half-lives and increased immunotoxicity risks [86,87]. These interspecies differences complicate the extrapolation of toxicity data from laboratory models to wildlife and human populations. Moreover, the combination of transgenerational epigenetic effects and immune modulation across species highlights the potential for dioxins to induce persistent health impacts that transcend generational and ecological boundaries. This underscores the necessity of adopting a One Health perspective, integrating human, animal, and ecosystem health to comprehensively address dioxin-related risks.

The variation in species sensitivity creates complex exposure scenarios where some wildlife species may serve as particularly sensitive sentinels for environmental contamination that can affect humans. Other species may accumulate high concentrations without obvious effects, potentially serving as hidden sources of contamination in food webs.

## 5. Human Health Implications

Human exposure to dioxins primarily occurs through the dietary intake of animal-derived fats, such as meat, dairy products, fish, and shellfish, due to their high lipid solubility and biomagnification through food chains [88,89,90]. Dietary exposure pathway directly connects human health to the health of livestock, fisheries, and wildlife, showing the fundamental interconnection central to the One Health paradigm. Contamination of animal feed, fishing grounds, or grazing areas directly translates to human exposure, while the same environmental reservoirs that affect wildlife populations are also potential sources of human dietary exposure. Although biomonitoring studies report declining background levels in many industrialized countries due to regulatory efforts [91,92], significant disparities persist. Vulnerable populations, such as Indigenous Arctic communities, subsistence fishers, and residents near informal e-waste sites, continue to experience disproportionate exposure burdens [25,30,93]. These environmental justice concerns reflect systemic inequities in exposure mitigation and access to safer food systems. Additional high-risk scenarios include occupational exposure (e.g., waste incineration, chemical manufacturing) and accidental releases near contaminated sites [94,95].

Biomonitoring of dioxin exposure traditionally relies on measurements in blood and adipose tissue, which reflect body burdens of these persistent compounds. Recent studies have documented declining background levels in many industrialized nations, largely due to regulatory interventions [91,92]. However, these sampling methods are invasive and may pose ethical or logistical challenges in large-scale or longitudinal studies. In this context, non-invasive biomarkers such as hair analysis have emerged as promising alternatives. Hair has been shown to correlate strongly with adipose tissue dioxin levels, with reported correlations reaching 95%, making it a valuable tool for population-based monitoring [96,97].

Key health concerns, primarily linked to dioxins and TEQ levels, include:

- **Carcinogenicity**: In 1997, based on animal and human epidemiology data, the International Agency for Research on Cancer (IARC) classified TCDD as a known human carcinogen [98]. This was supported by limited evidence in humans, sufficient evidence in experimental animals, and extensive mechanistic information, which was supported by subsequent evidence [99]. Other PCDDs, the non-chlorinated dibenzo-p-dioxin, and PCDFs were evaluated as not classifiable for their carcinogenicity to humans (Group 3) [100]. Epidemiological studies have linked high exposures (occupational, accidental) to increased risk of all cancers combined, as well as specific types like soft-tissue sarcoma and non-Hodgkin lymphoma [101,102,103].
- **Reproductive and Developmental Toxicity**: Prenatal and early-life exposure are critical concerns. Effects observed in humans include subtle developmental delays, altered thyroid hormone levels, effects on tooth development, potential impacts on sex ratio, and links to endometriosis [104,105,106]. Transgenerational effects via epigenetic modifications (e.g., DNA methylation changes) have been shown in animal models and are suspected in humans, potentially extending health impacts across generations [82].
- **Immunotoxicity**: Dioxins can suppress immune function, potentially increasing susceptibility to infections and impairing vaccine efficacy [107,108].
- **Endocrine Disruption**: Dioxins interfere with multiple endocrine systems, including thyroid, steroid hormone (estrogen, androgen), and insulin signaling pathways [109,110]. This can contribute to reproductive problems, metabolic disorders (e.g., diabetes risk), and potentially obesity [111].
- **Neurological Effects**: Developmental exposure to dioxins has been linked to cognitive and behavioral deficits in children, as well as impairments in motor function [112].
- **Other Adverse Effects**: Acute high exposure can result in chloracne, a severe skin condition [113]. Additionally, dioxins have been associated with cardiovascular and liver function disturbances [114].

These diverse human health effects run in parallel with many of the impacts observed in wildlife populations, including reproductive dysfunction, immune suppression, and developmental abnormalities. This similarity in health outcomes across species reinforces the mechanistic basis for One Health approaches and suggests that wildlife health data can inform human health risk assessment, while human health surveillance may provide insights relevant to wildlife conservation. However, the long biological half-life of dioxins in humans results in persistent body burdens, complicating risk assessment for chronic exposures at low doses [115].

## 6. Animal and Ecosystem Health Effects

Dioxins exert significant toxicity across a wide range of wildlife, particularly impacting species higher up the food chain and aquatic organisms [1]. The interconnection highlighted by One Health is evident here, as wildlife health reflects environmental contamination levels and can serve as an early warning for potential human health risks. Due to their position in food webs, many wildlife species have higher tissue concentrations than humans, making them valuable sentinels for detecting environmental pollution before it reaches levels that may affect human populations.

**Reproductive Failure and Developmental Abnormalities**: Classic examples include severe reproductive impairment (embryo mortality, deformities like crossed bills) and population declines in fish-eating birds (e.g., cormorants, terns) in the Great Lakes during the 1970s–1980s [116,117]. Similar effects, including reproductive dysfunction and skeletal abnormalities, have been observed in marine mammals (e.g., seals) in contaminated areas [118]. These wildlife reproductive failures served as early indicators of environmental contamination that simultaneously affected humans in the same regions.

**Immune Suppression and Increased Disease Susceptibility**: Dioxin exposure compromises immune function in fish, birds, and mammals, potentially increasing vulnerability to pathogens and other stressors [68,119]. Immune suppression in wildlife populations can facilitate the emergence and spread of infectious diseases that may also affect domestic animals and humans, particularly in cases of zoonotic pathogens.

**Endocrine Disruption in Wildlife**: Altered thyroid function, disrupted steroid hormone balance (leading to masculinization/feminization), and metabolic changes are documented across diverse taxa exposed to dioxins [120,121]. These endocrine disruption patterns suggest common mechanisms and provide opportunities for comparative research that benefits both wildlife conservation and human health protection.

**Bioaccumulation and Biomagnification**: Dioxins readily accumulate in fatty tissues and biomagnify significantly through aquatic and terrestrial food webs [122]. This puts apex predators (e.g., raptors, marine mammals like polar bears and orcas) at particularly high risk, as they accumulate contaminants from all lower trophic levels [123]. This biomagnification process directly connects environmental contamination to human food supplies, as many apex predators are also harvested for human consumption, particularly in subsistence communities.

**Ecosystem-Level Effects**: Beyond individual toxicity, dioxins can disrupt population dynamics, alter predator–prey relationships, and affect community structure [124]. Impacts on soil microbial communities involved in nutrient cycling and decomposition are also an area of research, potentially affecting essential ecosystem services [125,126,127]. Ecosystem-level disruptions can impact pollination, pest control, and soil formation, illustrating how ecosystem health directly supports human health and well-being.

**Wildlife as Sentinels**: Sensitive wildlife species act as crucial sentinels of environmental contamination. Monitoring dioxin levels and associated health effects (e.g., reproductive success, immune biomarkers) in indicator species provides valuable data on environmental quality and potential risks migrating up the food chain towards humans [128,129,130]. This highlights the importance of linking wildlife health data to human health risk assessment frameworks. Sentinel species monitoring can provide early warnings of contamination events, guide remediation priorities, and validate the effectiveness of pollution control measures across the human–animal–environment interface.

### 6.1. Wildlife Case Studies

**Seabirds**: Dioxins and related POPs are widespread in marine environments, where they accumulate throughout the food web, ultimately affecting top predators such as seabirds. Seabirds serve as effective bioindicators, reflecting the extent of environmental contamination in their habitats due to their position at the top of marine trophic chains [131]. Research has documented elevated concentrations of dioxins in seabird populations in regions such as the Baltic Sea and Canadian Arctic, highlighting their role in monitoring POP levels in marine ecosystems [132,133,134]. The contamination patterns observed in seabird populations directly inform human health risks for communities that consume marine resources from the same environments, particularly Arctic and coastal indigenous communities whose traditional diets rely heavily on marine foods.

**Coral Reefs**: Dioxins from coastal runoff inhibit coral gametogenesis, contributing to reef decline in the Great Barrier Reef [135,136,137]. Coral reef degradation affects the fisheries that many coastal communities depend upon for protein and economic security.

### 6.2. Microbial Remediation and Ecosystem Impacts

The degradation of highly chlorinated dioxins in contaminated environments relies on specialized microbial communities, with studies demonstrating that shifts in microbial population structure and metabolic activity are critical for breaking down these persistent pollutants [138]. Controlled batch microcosm experiments reveal that tailored microbial consortia—particularly those enriched with dioxin-degrading bacteria like Bacillus species—drive efficient dechlorination and mineralization processes [126,139]. This aligns with bioremediation strategies such as microbial seeding, where introducing targeted strains or consortia (e.g., *Bacillus* sp. SS2 or *Comamonas* sp. KD7) enhances degradation rates by up to 94.9% in contaminated soils [139,140]. Recent advances highlight the potential of combining bioaugmentation with phytoremediation or composting to optimize microbial activity and improve cleanup outcomes in field applications [126]. These bioremediation approaches show how ecosystem processes can benefit both environmental health and human health outcomes.

## 7. One Health Perspective on Dioxin Exposure Management

The One Health framework offers a structured approach to integrate the complex environmental, animal, and human dimensions of dioxin contamination [141]. The systems-based approach shown in Figure 1 demonstrates how dioxin contamination creates shared vulnerabilities across environmental, animal, and human health domains, necessitating coordinated responses that address all three simultaneously. This integration is not merely conceptual but reflects the fundamental biological and ecological reality that dioxin exposure pathways cross traditional disciplinary boundaries. Figure 1 illustrates that effective management requires addressing these interconnections rather than treating them as separate issues. For example, agricultural soil contamination affects crop production, livestock health, and human dietary exposure simultaneously, while also affects soil organisms and terrestrial wildlife that use agricultural landscapes.

### 7.1. Integrated Surveillance Systems: Moving Beyond Siloed Monitoring

Integrated surveillance is a cornerstone of the One Health approach to dioxin management, requiring the combination of multiple data streams to provide a comprehensive view of contamination patterns and health risks. Environmental monitoring, including air, water, soil, and sediment sampling, tracks contamination at its source and in ecosystems. Food safety surveillance measures help detect dioxin levels in animal-derived products, such as meat, milk, eggs, and fish, thereby preventing human exposure to these contaminants through the diet. Wildlife biomonitoring, particularly in sentinel species, serves as an early warning system for ecological impacts and the health of ecosystems [142]. Human biomonitoring, through the measurement of dioxins in blood, adipose tissue, or non-invasive matrices like hair, detects exposures in both general and high-risk populations. An emerging complement to formal surveillance systems is citizen science, which empowers local communities (such as farmers or fishers) to report unusual animal health events or environmental changes. Digital platforms and mobile applications can facilitate these reports, creating real-time connections between community observations and scientific monitoring. By integrating these diverse surveillance strategies, the One Health framework facilitates the early detection of contamination hotspots, identifies emerging trends, and supports rapid and coordinated responses across sectors [143,144,145,146].

**Global Biobanks**: The One Health Biobank Initiative (OHBI) archives environmental, animal, and human samples for retrospective analysis of dioxins, facilitating transdisciplinary research [147].

### 7.2. Transdisciplinary Risk Assessment

Traditional risk assessments often focus on a single species (humans) or pathway. The One Health approach encourages collaborative risk characterization involving toxicologists, ecologists, epidemiologists, veterinarians, physicians, social scientists, and policymakers. It considers multiple exposure pathways, cumulative exposures (mixtures), varying species sensitivities, ecosystem impacts, and socio-economic factors. This integrated approach recognizes that dioxin exposure rarely occurs in isolation but as part of complex environmental mixtures that affect multiple species simultaneously through interconnected pathways [20,148].

Transdisciplinary risk assessment also incorporates traditional ecological knowledge from indigenous and local communities, whose observations of environmental and wildlife health changes can provide early indicators of contamination problems. This community-based knowledge complements scientific monitoring data and helps to identify vulnerable populations and ecosystems that might otherwise be overlooked.

### 7.3. Cross-Sectoral Coordinated Response

Dioxin contamination events (e.g., feed/food crises) require rapid, coordinated action involving environmental agencies, agricultural departments, food safety authorities, public health bodies, and veterinary services. One Health fosters the necessary interagency communication and pre-established response protocols. The Belgian dioxin crisis of 1999 demonstrated both the consequences of inadequate coordination and the benefits of integrated response systems, leading to major reforms in EU food safety regulations that now serve as models for coordinated One Health approaches [141,149].

Effective coordinated response requires not only inter-agency cooperation but also engagement with affected communities, industry stakeholders, and international partners, particularly given the transboundary nature of dioxin contamination.

### 7.4. Knowledge Translation and Co-Production

Effective management requires collaboration between researchers generating scientific evidence and the stakeholders (policymakers, industry, communities) who use it. The One Health approach encourages co-design of research questions and interventions, ensuring relevance and facilitating uptake. This participatory approach recognizes that local communities, farmers, fishers, and other stakeholders possess valuable knowledge about environmental changes and health patterns that can help scientific research, while scientific findings must be translated into actionable guidance that stakeholders can implement [150,151].

### 7.5. Risk Communication and Public Engagement

Communicating complex risks associated with invisible contaminants like dioxins requires transparency and tailored messaging for different audiences (public, health professionals, farmers, etc.). The One Health framework supports consistent messaging across sectors and engages communities in understanding risks and participating in solutions. Effective risk communication must acknowledge the interconnected nature of dioxin exposure risks, helping communities understand how environmental contamination affects both their own health and the health of animals and ecosystems they depend upon [152]. Public engagement strategies should also recognize that many communities have direct relationships with potentially affected wildlife and ecosystems, creating opportunities for community-based monitoring and stewardship that can complement formal surveillance systems.

### 7.6. Economic Considerations

Dioxin contamination incurs significant economic costs (remediation, healthcare, lost trade, food recalls—e.g., Belgian crisis) [153]. A One Health economic assessment integrates these costs alongside health and environmental impacts to demonstrate the value of preventative measures and inform cost-effective policy choices. Economic analysis should account for the interconnected nature of impacts, recognizing that environmental contamination simultaneously affects healthcare costs, agricultural productivity, wildlife conservation costs, and the value of ecosystem services.

### 7.7. Policy Innovations

Policy innovations play a critical role in dioxin risk management under the One Health framework. These range from targeted sectoral regulations to broader interagency governance models. For example, the European Union’s 2022 Waste Framework Directive mandates dioxin screening in recycled plastics, specifically to prevent contamination from re-entering the food chain through food packaging materials [154]. At a broader scale, operational models such as the WHO-FAO-OIE (now WOAH) Tripartite collaboration, recently expanded into the Quadripartite with the inclusion of UNEP, offer templates for coordinated action across sectors. While initially designed for zoonotic disease control, these collaborative structures are increasingly recognized as adaptable for managing environmental contaminants, such as dioxins [141]. Beyond regulatory limits and technological controls, public health guidance represents a critical layer of policy innovation. In the European Union, for example, consumer information campaigns have been launched in certain Baltic Sea regions to mitigate dietary exposure. These initiatives, supported by regulations such as EU 915/2023, advise vulnerable groups (e.g., pregnant women, children) to consume fish frequently for its nutritional benefits while recommending the selection of smaller, younger fish from species lower in the food web to avoid high dioxin accumulation. Furthermore, public health agencies provide guidance on food preparation, such as grilling or baking fish to allow fats containing dioxins to drip away, thereby reducing intake.

Table 1 provides concrete examples of integrated strategies across environmental, human, wildlife, and policy domains, illustrating practical applications of the One Health framework for dioxin risk reduction and highlighting both implementation successes and ongoing challenges. These examples show how effective dioxin management requires coordinated action across traditional sectoral boundaries.

## 8. Case Studies and Policy Responses

### 8.1. High-Profile Incidents Underscore the Need for Integrated Approaches

**Seveso Disaster (Italy, 1976)**: This industrial accident resulted in the release of several kilograms of dioxins, leading to extensive environmental contamination and acute human health effects, including chloracne. Long-term epidemiological studies in the exposed cohort have since identified increased risks of cancer, as well as metabolic and reproductive disorders [157,158,159]. It catalyzed the EU’s Seveso Directives on industrial accident prevention, demonstrating the long legacy of environmental contamination on human health. While extensive human and environmental data exist, integrated One Health analyses, incorporating parallel wildlife or domestic animal health monitoring from the same time, are less developed.

**Belgian Dioxin Crisis (1999)**: The contamination of animal feed with PCBs and dioxins resulted in widespread contamination of meat, dairy products, and eggs. It caused massive economic losses, undermined consumer confidence, and revealed critical failures in food chain surveillance and cross-sectoral communication [160,161]. That crisis revealed how food chain contamination simultaneously affects animal health, human health, and economic systems, leading to major reforms in EU food safety regulations that now serve as models for integrated One Health approaches. The incident highlighted the vulnerability of interconnected food systems and the economic consequences of inadequate cross-sectoral coordination. Spurred major reforms in EU food safety regulations and traceability, including strengthening EFSA. Highlights the vulnerability of the food chain and the economic consequences of contamination.

**Agent Orange (Vietnam War, 1961–1971)**: An herbicide mixture contaminated with TCDD was sprayed extensively. It resulted in persistent environmental “hotspots”, long-term ecological damage, and ongoing debate and research into health effects (cancers, birth defects, neurological disorders) in Vietnamese populations and exposed veterans [162,163,164]. It illustrates the devastating long-term environmental and multi-generational health impacts of large-scale contamination. Like Seveso, the focus has been predominantly on human health and environmental levels, with fewer integrated studies assessing concurrent impacts on wildlife or livestock health in affected zones. A more integrated One Health analysis could provide insights into the full ecosystem impacts and inform more comprehensive restoration strategies.

**Yushō and Yucheng Incidents (Japan, 1968; Taiwan, 1979)**: These incidents involved mass poisonings resulting from cooking oil contaminated with PCBs and PCDFs. Long-term follow-up documented severe skin lesions, immune dysfunction, adverse reproductive outcomes, and developmental effects in children born to exposed mothers [165,166]. Yusho and Yucheng incidents demonstrated how food chain contamination can cause acute health crises, while also creating long-term health surveillance challenges.

### 8.2. More Recent Contamination Incidents

While historical cases provide foundational lessons, more recent incidents underscore the ongoing and evolving nature of dioxin contamination. The collapse of the World Trade Center in 2001 resulted in intense, long-burning fires that released dioxins contained in the toxic dust cloud, highlighting the risk of acute, large-scale urban emissions. In another vein, a series of food and feed contamination events in the 2000s, including contaminated guar gum in India (2007), dioxin in buffalo milk for mozzarella production in Italy (2007–2008), and PCB/dioxin-contaminated pork in Ireland (2008), revealed persistent vulnerabilities in global supply chains, particularly related to feed processing and auxiliary chemicals. Moreover, a 2019 incident in an Indonesian village, where plastic waste was burned as fuel for local tofu production, led to severe dioxin contamination of foodstuffs, with levels comparable to those in Agent Orange hotspots. This case exemplifies a modern source–pathway–receptor chain, linking the global plastic waste crisis to localized, high-level food contamination in informal economic settings.

### 8.3. Policy Responses

**International**: The Stockholm Convention on Persistent Organic Pollutants (POPs) is the key global treaty. It mandates parties to reduce or eliminate the release of unintentionally produced POPs, such as dioxins, primarily through National Implementation Plans (NIPs) that require the use of Best Available Techniques (BAT) and Best Environmental Practices (BEP) for key source categories [167].

**National/Regional**: Many countries/regions have implemented stringent regulations, including the following:

**Emission Limits**: Strict limits on dioxin emissions from sources like incinerators and industrial plants (e.g., EU Industrial Emissions Directive, 2010).

**Food/Feed Safety**: Maximum residue limits (MRLs) or action levels for dioxins (PCDD/PCDFs and dl-PCBs, often expressed as TEQ) in various food and feed categories [6,168]. Regular monitoring programs are essential for enforcement.

**Contaminated Sites**: Soil cleanup standards and remediation guidelines (e.g., US EPA Superfund program) [169].

**Health Guidance**: The World Health Organization (WHO) has established Tolerable Intake levels to guide risk assessment and regulatory action [90].

### 8.4. Global Policy Alignment

The EU Plastic Regulation Act now enforces real-time dioxin sensors in waste facilities, reducing contamination incidents by enabling rapid detection and response [156]. This is an example of how technological innovation can support integrated environmental and health protection by preventing contamination at the source.

## 9. Challenges and Future Directions

Addressing dioxin contamination globally presents ongoing challenges, yet a One Health approach offers promising solutions. Limited monitoring capacity in Low- and Middle-Income Countries (LMICs) is a major challenge, driven by the high cost of dioxin analysis, the need for high-resolution analytical equipment, and specialized technical expertise. These limitations create significant data gaps that affect the understanding of global contamination patterns and their health impacts across regions, highlighting the need for capacity building through training, technology transfer, and international cooperation initiatives [170].

A further complicating factor is the ongoing identification of novel dioxin-like compounds (DLCs) beyond the traditional PCDD/Fs and dl-PCBs. These include certain brominated and mixed halogenated dibenzo-p-dioxins and furans (PBDD/Fs, PXDD/Fs), as well as other structurally diverse aryl hydrocarbon receptor (AhR) agonists. Many of these compounds exhibit similar toxicological properties but are not consistently monitored or included in the current Toxic Equivalency Factor (TEF) framework. Their presence in consumer products, such as certain plastics and electronic waste, and in environmental matrices creates a “blind spot” in risk assessment. Addressing this challenge requires updating regulatory definitions, expanding monitoring programs to include these emerging DLCs, and funding research to establish their relative potencies and health impacts to determine if they should be formally classified as POPs under international treaties.

Emerging sources, such as informal e-waste recycling and biomass combustion, alongside climate-driven remobilization from permafrost and interactions with microplastics, necessitate targeted interventions that consider their impacts across environmental, animal, and human health domains simultaneously. These emerging challenges require adaptive management approaches that can respond to changing exposure patterns and novel contamination pathways.

Assessing mixture effects remains complex, requiring advanced toxicological models, including omics and computational approaches that can account for the complex interactions between dioxins and other environmental contaminants to which organisms are simultaneously exposed [171,172]. Furthermore, wildfire intensification due to climate change may remobilize soil-bound dioxins, requiring the integration of fire management and food safety surveillance to protect both wildlife habitats and human food supplies. This demonstrates how climate change adaptation strategies must consider pollution management to be fully effective [173]. Emerging technologies, such as CRISPR-based biosensors, enable on-site dioxin detection in LMICs, addressing cost and expertise barriers while providing rapid feedback for integrated surveillance systems [174,175].

Uneven regulatory enforcement creates pollution havens, indicating the need for strengthened international cooperation and technical assistance to ensure that environmental health protection measures are globally implemented. The cleanup of legacy contamination is resource-intensive and benefits from innovative technologies like biochar amendment and phytoremediation, as well as public–private partnerships that can mobilize the resources needed for large-scale remediation efforts.

Future directions include developing global surveillance platforms that integrate environmental, food, wildlife, and human biomonitoring data using geospatial tools and artificial intelligence for real-time trend detection. These platforms should be designed to accommodate diverse data types and sources while providing accessible interfaces for different user communities, from researchers to policymakers to community organizations [155,176]. Such platforms could be operationalized by building upon existing infrastructure, such as the WHO’s Global Environment Monitoring System (GEMS) or the Digital Observatory for Nature and Health. Additional priorities should include validating sensitive biomarkers, such as epigenetic marks and metabolomics profiles, for early exposure detection across species, with standardized non-invasive methods like hair analysis that can be applied to both human and wildlife populations.

Climate-resilient strategies, including enhanced soil stabilization in permafrost regions and improved waste management in areas prone to extreme weather events, should be integrated into climate adaptation frameworks to prevent climate change from exacerbating dioxin exposure risks. Concrete measures include integrating dioxin screening into national climate adaptation plans, supporting the transition to controlled waste incineration over open burning, and funding research on soil stabilization techniques in vulnerable permafrost regions. Community engagement through citizen science and participatory monitoring addresses environmental justice concerns while building local capacity for ongoing surveillance and response [25]. The integration of climate resilience into One Health strategies, such as permafrost stabilization projects that consider both wildlife habitat protection and human community safety, and microplastic filtration systems that protect both marine ecosystems and seafood safety, is essential to mitigating future risks. Global policy harmonization, supported by Stockholm Convention funding mechanisms and UNEP’s Pollution-Free Planet Initiative, alongside scalable remediation technologies and transdisciplinary One Health training programs that prepare the next generation of professionals to work across traditional disciplinary boundaries, will foster collaborative solutions to mitigate dioxin impacts effectively [177,178]. Successful implementation will depend on clear implementation pathways, such as leveraging the Stockholm Convention’s financial mechanism to support LMICs and formally expanding the mandate of the Quadripartite Alliance (FAO, UNEP, WHO, WOAH) to explicitly include coordinated action on persistent chemical pollutants.

## 10. General Overview and Final Considerations

Dioxins represent a significant and persistent threat to integrated planetary health systems through their bioaccumulative properties and toxicological impacts across trophic levels. The One Health framework provides a critical interdisciplinary platform for addressing these complex environmental contaminants by facilitating synergistic collaboration among environmental toxicologists, veterinary scientists, human health practitioners, and ecological researchers. Given the interconnected nature of dioxin exposure pathways and health impacts across species and ecosystems, this collaborative approach is not merely beneficial but necessary.

Toxicological advances revealing the mechanistic interactions between dioxins and cellular signaling pathways, particularly the aryl hydrocarbon receptor (AhR), underscore the need for comparative species-specific risk assessments that account for taxon-dependent sensitivity variations while recognizing the underlying mechanistic similarities that enable cross-species extrapolation. This mechanistic understanding provides the scientific foundation for One Health approaches by demonstrating both the commonalities and differences in how dioxins affect different species.

Emerging technologies, including high-resolution analytical chemistry, non-targeted screening approaches, and computational toxicology, offer opportunities for comprehensive exposure characterization, which can be applied across environmental, wildlife, and human health domains. Meanwhile, sustainable remediation technologies utilizing enhanced bioremediation and photocatalytic degradation present promising intervention strategies that can simultaneously benefit ecosystem health and human health outcomes.

The implementation of effective dioxin management faces substantial challenges, including fragmented monitoring networks, regulatory inconsistencies across jurisdictions, and the effects of climate-induced environmental changes on dioxin mobilization patterns. These obstacles can only be overcome through coordinated global governance frameworks that transcend traditional disciplinary boundaries and geopolitical constraints, supported by capacity building initiatives that strengthen local expertise in all regions.

Finally, protecting the intricate web of interconnected health systems from dioxins’ persistent toxic legacy requires a transformative shift toward integrative One Health approaches, embracing methodological pluralism, promoting knowledge co-production across sectors, and recognizing the fundamental interdependence of environmental integrity and holistic health outcomes. This paradigmatic evolution in addressing complex environmental contaminants, such as dioxins, may serve as a model for managing other persistent pollutants that threaten planetary health, providing a template for the collaborative, interdisciplinary approaches needed to address the complex environmental health challenges of the 21st century.

## 11. Conclusions

Dioxins remain urgent global health risks due to their persistence, bioaccumulation, and toxicity to humans, wildlife, and ecosystems. Effective management requires coordinated One Health approaches, integrated surveillance, and evidence-based policies bridging disciplines. Climate changes and new sources, such as microplastics, intensify exposure challenges, making adaptive and harmonized action essential. Capacity building, improved stakeholder engagement, and regulatory consistency are critical for long-term prevention and control.

## Figures and Tables

**Table 1:** Integrated strategies for dioxin risk reduction.

Domain	Intervention	Example	Effectiveness/Success Indicators	Limitations
Environmental	Enhanced biochar soil remediation	Optimized biochar amendment using carrier gas injection during pyrolysis effectively reduces dioxin bioavailability by limiting oxygen contact, achieving up to 65% reduction in soil dioxin concentrations [63,64].	Quantifiable reduction in soil dioxin concentrations (target: >50%); decreased bioavailability to soil organisms; improved plant uptake profiles	Applicable to contaminated agricultural and urban soils; requires site-specific optimization and long-term stability monitoring
Environmental	Integrated phytoremediation systems	*Trifolium repens* combined with specialized microbial consortia (*Comamonas* sp.) enhances dioxin degradation through synergistic plant-microbe interactions [126,140].	Measured dioxin degradation rates in contaminated soil; plant survival and growth metrics; microbial community stability assessments	Environmentally sustainable and cost-effective; slower remediation timeline; effectiveness varies with soil conditions and climate; requires ongoing maintenance
Human Health	Community-based hair biomonitoring programs	Non-invasive hair analysis demonstrates 95% correlation with adipose tissue dioxin levels, enabling population-wide exposure assessment in vulnerable communities [96,155].	Statistical correlation with traditional biomarkers; community participation rates (target: >70%); early detection of exposure trends	Highly useful for population studies; requires standardized collection and analysis protocols; culturally acceptable across diverse communities; lower cost than invasive sampling
Wildlife	Multi-species sentinel monitoring networks	Coordinated monitoring of Baltic Sea seabirds, fish, and marine mammals providing integrated assessment of ecosystem contamination and early warning for human health risks [131,142].	Temporal trends in tissue concentrations; reproductive success metrics; correlation with environmental contamination levels	Effective for ecosystem-scale monitoring; requires long-term commitment and taxonomic expertise; species selection must consider ecology and logistics; valuable for early warning systems
Policy	Circular economy enforcement with real-time monitoring	EU regulations mandating continuous dioxin sensors in waste processing facilities, coupled with recycled material screening to prevent re-entry into food packaging supply chains [154,156].	Reduction in contamination incidents; compliance rates with detection requirements; decreased dioxin levels in recycled materials	High potential for global adoption; requires initial technology investment; enables rapid response to contamination events; supports sustainable materials management
Policy	Community-based digital surveillance systems	Mobile applications enabling farmers, fishers, and community members to report livestock health anomalies, unusual wildlife mortality, or environmental changes linked to potential dioxin contamination [145,146].	Number and quality of community reports; correlation between reports and confirmed contamination events; response time to reported incidents	Promotion of environmental justice and community engagement; requires digital literacy and infrastructure; builds local monitoring capacity; enables rapid detection in remote areas

## Data Availability

No new data were created or analyzed in this work. Data sharing is not applicable to this article.
